# Trends in Myopia Development Among Primary and Secondary School Students During the COVID-19 Pandemic: A Large-Scale Cross-Sectional Study

**DOI:** 10.3389/fpubh.2022.859285

**Published:** 2022-03-22

**Authors:** Jingfeng Mu, Haoxi Zhong, Meizhou Liu, Mingjie Jiang, Xinyi Shuai, Yanjie Chen, Wen Long, Shaochong Zhang

**Affiliations:** ^1^Shenzhen Eye Hospital, Shenzhen Eye Institute, Shenzhen Eye Hospital affiliated to Jinan University, Shenzhen, China; ^2^School of Optometry, Shenzhen University, Shenzhen, China

**Keywords:** coronavirus, epidemic, myopia, spherical equivalent refraction, home confinement

## Abstract

**Objectives:**

To evaluate myopia development among primary and secondary school students during the coronavirus 2019 (COVID-19) pandemic.

**Methods:**

A cross-sectional study was conducted to evaluate the development of myopia among students in Shenzhen, China during the COVID-19 outbreak.

**Results:**

The study included 1,472,957 and 1,573,824 students in 2019 and 2020, respectively. The prevalence of myopia was 46.9 and 50.5% in 2019 and 2020, respectively. The prevalence of myopia among students in the former Shenzhen Special Economic Zone (SEZ) was higher than that in areas outside the former Shenzhen SEZ (2019: 47.0 vs. 43.7%; 2020: 50.5 vs. 47.3%). The prevalence of myopia among girls was higher than that among boys (2019: 50.4 vs. 44.0%; 2020: 54.0 vs. 47.6%). The 50th percentile (*P*_50_) of spherical equivalent refraction (SER) in the right eye among girls was lower than that in boys. The prevalence of myopia continued to increase as the grade increased, with the greatest annual increase observed in Grades 2–5 (3.4–3.9%). The *P*_50_ of SER in the right eye of students decreased as the grade increased.

**Conclusions:**

The prevalence of myopia among students increased during the COVID-19 pandemic, especially in primary school Grades 2–5.

## Introduction

The coronavirus 2019 (COVID-19) pandemic started in China at the end of 2019. To curb the spread of the pandemic, many countries, including China, implemented measures such as crowd limitation and social distancing (1, 2). The COVID-19 outbreak coincided with the Chinese elementary and middle school winter vacation, and the start of the spring term was delayed nationwide until the end of May 2020. Approximately 278 million primary and secondary school students countrywide were confined to their homes and received online tuition (3, 4). During home confinement, the time spent playing games, chatting online, and browsing the internet increased substantially.

Although home confinement is an important measure taken to reduce social interaction and control disease spread during public health emergencies, long-term school closures and home-based learning may have an impact on the eye health of children and adolescents. Myopia is a serious health concern among children and adolescents in China. Studies have shown that mild and moderate myopia affect at least 25% of Europeans and North Americans, 5% of Africans, and ~80% of East Asians (5–10). The prevalence of myopia in China is the highest in the world, and its prevalence among elementary and middle school students is estimated to be 59.4–82.5% (11); it is a major problem that has an adverse effect on students' the physical and mental health. The prevalence of myopia in China continues to rise, and the age of onset is decreasing. It is imperative to raise awareness and take effective measures to curb the onset and progression of myopia.

A previous study found that each additional hour of outdoor activity per week reduced the risk of myopia by 2% (12). As the duration of outdoor activity increased from <5 h per week to 14 h per week, the probability of myopia decreased by one-third (13). According to a study in Chongqing, China, 97.7% of primary and secondary school students used computers and phones to study online during the COVID-19 pandemic (14). The use of electronic devices may accelerate the development of myopia and increase the risk of myopia in children and adolescents (15). Attention should thus be paid to students' visual health during the COVID-19 pandemic. The purpose of this study was to determine the trend in myopia among elementary and middle school students during the COVID-19 pandemic and to investigate the effect of home confinement on the development of myopia.

## Methods

### Study Population

Shenzhen is China's first special economic zone, located at the forefront of the Pearl River Delta, forming a bridge between Hong Kong and Mainland China. It has direct jurisdiction over nine districts and one new district. Four districts of Shenzhen (Nanshan District, Futian District, Luohu District, and Yantian District) are located in the former Shenzhen Special Economic Zone (SEZ), and the other five districts (Bao'an District, Guangming District, Longhua District, Pingshan District, and Longgang District) and Dapeng New District are located outside the former Shenzhen SEZ. A total of 1,472,957 students from all primary and secondary schools (757 schools) in Shenzhen underwent eye examinations from September 1 to November 30, 2019, an effective participation rate of 95.3%. A total of 1,573,824 students from all primary and secondary schools (782 schools) in Shenzhen were examined for eye health from September 1 to November 30, 2020, with an effective participation rate of 97.8%.

This study was approved by the Ethics Committee of the Shenzhen Eye Hospital. Parents or guardians of the students signed an informed consent form.

### Visual Acuity Test

The examiners instructed students who were wearing glasses or contact lenses to remove them prior to examination. The students were advised not to squint, peek, rub, lean forward, or follow instructions from others. In this study, students were examined using an electronic logarithmic visual acuity chart (Eye Vision 1603-01; Guangdong Eye Vision Medical Technology Co., Ltd, Guangzhou, China).

### Refraction Test

The refraction test was performed using an autorefractor (NIDEK AR-1; NIDEK Co., Ltd., Tokyo, Japan) without ciliary muscle paralysis. Before daily screening, the instrument was calibrated using standard analog eyes and the cylindrical lens was adjusted to negative values. Each student had the refraction automatically measured three times in each eye using an autorefractor, which then provided the average value. Students wearing glasses were instructed to remove them before the test was performed, and those wearing contact lenses were instructed to remove their lenses more than 30 min before the refraction test was performed.

### Definition of Myopia

Spherical equivalent refraction (SER) was calculated using the cylindrical degree and spherical degree as follows:


$$\begin{array}{l} \text{SER }=\text{ cylindrical degree }\times \;0.5\;+\text{ spherical degree}. \end{array}$$

According to the specification for screening of refractive error in primary and secondary school students (WS/T 663-2020), myopia was defined as uncorrected visual acuity of any eye <5.0 with SER < −0.50 D; those who wore an orthokeratology lens were also defined as myopic (16). Students whose SER range was −3.00 D ≤ SER < −0.50 D were defined as mildly myopic, those with SER range −6.00 D ≤ SER < −3.00 D were defined as moderately myopic, and those with SER < −6.00 D were defined as highly myopic.

### Statistical Analyses

Statistical analysis was performed using R software version 4.1.0 (R Foundation for Statistical Computing, Vienna, Austria), and statistical significance was set at *p* < 0.05. The prevalence of myopia among different groups was compared using the chi-squared test. The Kolmogorov–Smirnov test was performed to verify the normality of the data. As the SER of primary and secondary school students does not follow a normal distribution (*D* = 0.148, *p* < 0.05), the 50th percentile (*P*_50_) of the SER was used to represent the concentration trend, and the 25th and 75th percentiles were used to represent discrete trends. Spearman's rank correlation coefficient was used to assess the correlation between the SER of the left and right eyes. As the SER of the two eyes were highly correlated (Spearman's rank correlation = 0.859, *p* < 0.05), and we used the SER of the right eye of the students as the basis for evaluating the development of myopia.

## Results

A total of 1,472,957 students (807,664 boys and 665,293 girls) from Grade 1 of primary school to Grade 12 of high school had their visual acuity and refraction tested in 2019, and the prevalence of myopia was 46.9% (690,129/1,472,957). A total of 1,573,824 students (859,931 boys and 713,893 girls) from Grade 1 of primary school to Grade 12 of high school had their visual acuity and refraction tested in 2020, and the prevalence of myopia was 50.5% (794,889/1,573,824).

**Figure 1:**
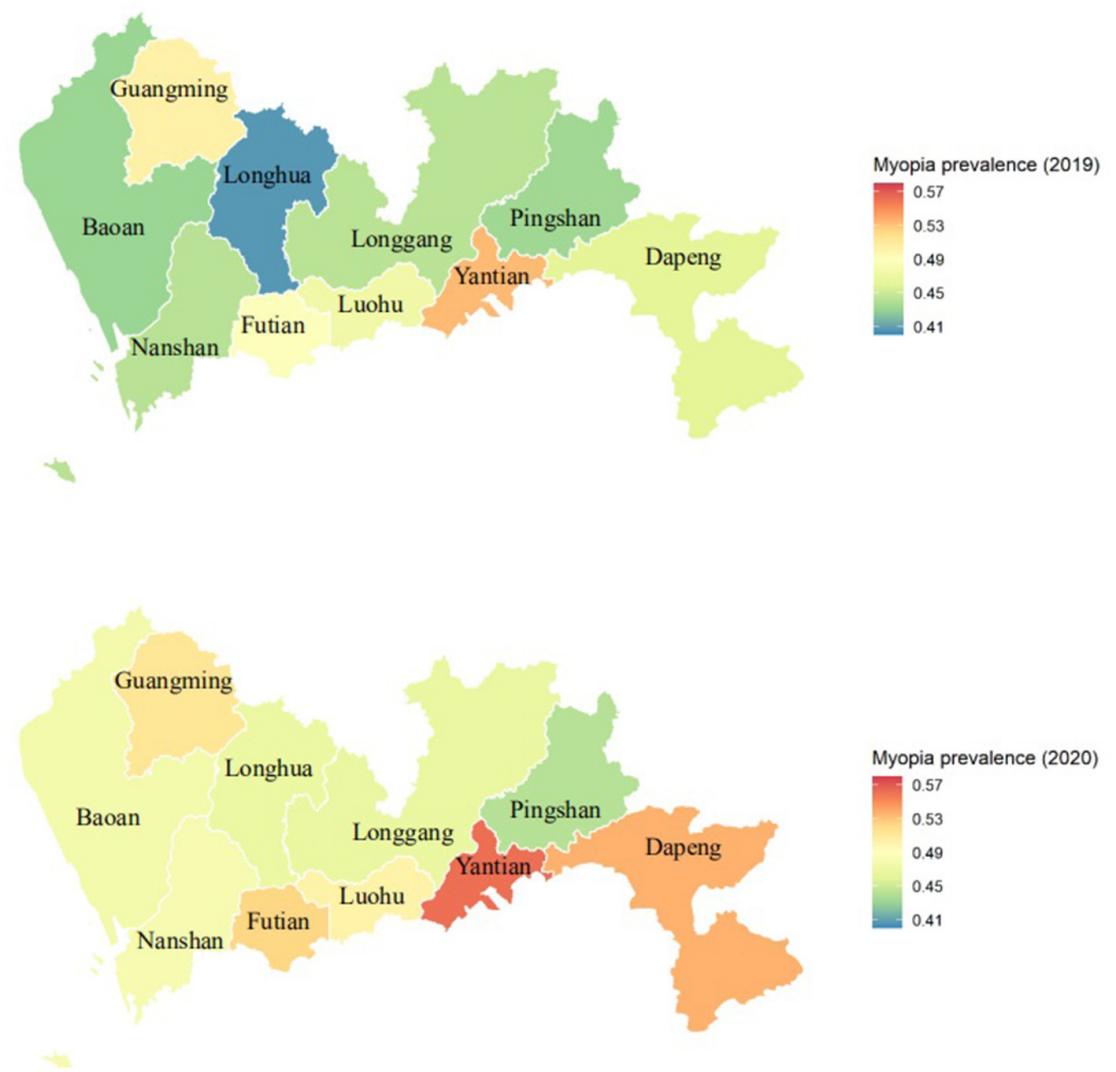
The spatial distribution map of the prevalence of myopia in elementary and middle school students in Shenzhen, China.

The prevalence of myopia among elementary and middle school students in all administrative districts of Shenzhen was higher in 2020 than in 2019 (*p* < 0.05). The prevalence of myopia among elementary and middle school students in areas in the former Shenzhen SEZ was higher than that in areas outside the former Shenzhen SEZ from 2019 to 2020. The prevalence of myopia in Dapeng New District had the greatest increase, with an 7.6% increase of from 46.1% in 2019 to 53.7% in 2020, and Pingshan District had the smallest increase, with an 0.8% increase from 43.3% 2019 to 44.1% in 2020 (Table 1). According to the spatial distribution map of the prevalence of myopia among elementary and middle school students in Shenzhen, the prevalence of myopia was highest in Yantian District (Figure 1).

**Table 1:** The prevalence of myopia among primary and secondary school students in Shenzhen, China according to their demographic characteristics.

**Characteristics**	**2019**		**2020**		***χ^2^***	***P***	**Prevalence_**2020**_-Prevalence_**2019**_**
	***n***	**Prevalence**	***n***	**Prevalence**			
Areas within the former Shenzhen SEZ	455,218	0.470	495,047	0.505	1,162.8	<0.001	0.035
Nanshan District	159,244	0.442	167,226	0.479	449.5	<0.001	0.037
Yantian District	23,526	0.534	27,171	0.562	40.0	<0.001	0.028
Luohu District	128,710	0.473	131,875	0.502	219.3	<0.001	0.029
Futian District	143,738	0.488	168,775	0.523	380.4	<0.001	0.035
Areas outside the former Shenzhen SEZ	1,017,739	0.437	1,078,777	0.473	2,736.5	<0.001	0.036
Dapeng New District	13,838	0.461	16,552	0.537	174.1	<0.001	0.076
Longgang District	358,456	0.443	370,568	0.467	423.2	<0.001	0.024
Pingshan District	53,259	0.433	58,312	0.441	7.2	0.007	0.008
Bao'an District	350,526	0.431	364,840	0.475	1,396.6	<0.001	0.044
Longhua District	170,031	0.407	183,930	0.468	1,335.2	<0.001	0.061
Guangming District	71,629	0.503	84,575	0.513	15.5	<0.001	0.010
**Grades**							
Grade 1	197,708	0.138	191,089	0.138	0.0	0.999	0.000
Grade 2	194,618	0.188	199,144	0.222	697.5	<0.001	0.034
Grade 3	173,369	0.287	193,208	0.324	588.6	<0.001	0.037
Grade 4	162,827	0.405	170,953	0.444	519.2	<0.001	0.039
Grade 5	156,464	0.519	161,082	0.556	437.1	<0.001	0.037
Grade 6	147,307	0.622	152,327	0.644	156.1	<0.001	0.022
Grade 7	113,963	0.687	133,986	0.712	183.5	<0.001	0.025
Grade 8	105,439	0.745	115,474	0.771	203.4	<0.001	0.026
Grade 9	88,408	0.784	99,468	0.808	166.6	<0.001	0.024
Grade 10	55,263	0.828	66,201	0.840	31.4	<0.001	0.012
Grade 11	48,675	0.837	58,153	0.850	34.1	<0.001	0.013
Grade 12	28,916	0.868	32,739	0.866	0.5	0.459	−0.002
**Sex**							
Boys	807,664	0.440	859,931	0.476	2,174.0	<0.001	0.036
Girls	665,293	0.504	713,893	0.540	1,788.9	<0.001	0.036

Except for the students in Grades 1 and 12, the prevalence of myopia among students in other grades increased to varying degrees from 2019 to 2020 (*p* < 0.05). Primary school students in Grade 4 had the largest increase in myopia prevalence (3.9%), followed by those in Grades 2, 3, and 5, all of which had an increase in prevalence of >3.0%.

**Figure 2:**
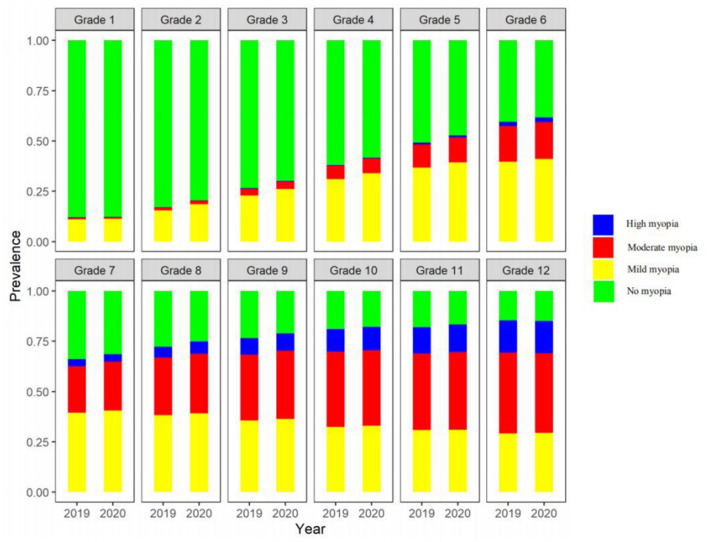
The prevalence of myopia in primary and secondary school students according to grade.

The prevalence of myopia in boys was 44.0 and 47.6% in 2019 and 2020, respectively, while that in girls was 50.4 and 54.0%, respectively. The prevalence among girls was higher than among boys in both 2019 and 2020. The prevalence of myopia increased with grade, with a higher increase (3.4–3.9%) in Grades 2 to 5 in primary school than in other grades, as shown in Table 1. Chi-square tests for trend revealed that the prevalence of myopia increased significantly with increasing grade in 2019 (χ^2^ = 338,149.1, *p* < 0.001) and in 2020 (χ^2^ = 355,556.3, *p* < 0.001) (Figure 2; Table 2). As shown in Table 2, the prevalence of high myopia and moderate myopia increased with increasing grade, while that of mild myopia increased with increasing grade from Grades 1 to 6, and gradually decreased with increasing grade from Grades 7 to 12.

**Table 2:** The prevalence of myopia of primary and secondary school students in Shenzhen, China according to school grade.

**Grades**	**2019**				**2020**			
	**No myopia**	**Mild myopia**	**Moderate myopia**	**High myopia**	**No myopia**	**Mild myopia**	**Moderate myopia**	**High myopia**
Grade 1	0.862	0.127	0.009	0.002	0.862	0.130	0.007	0.002
Grade 2	0.812	0.172	0.014	0.002	0.778	0.204	0.016	0.002
Grade 3	0.713	0.250	0.034	0.003	0.676	0.285	0.036	0.003
Grade 4	0.595	0.335	0.065	0.005	0.556	0.365	0.073	0.005
Grade 5	0.481	0.395	0.114	0.010	0.444	0.421	0.124	0.011
Grade 6	0.378	0.424	0.177	0.021	0.356	0.438	0.184	0.022
Grade 7	0.313	0.421	0.230	0.036	0.288	0.431	0.244	0.037
Grade 8	0.255	0.403	0.287	0.055	0.229	0.414	0.297	0.060
Grade 9	0.216	0.376	0.326	0.082	0.192	0.383	0.339	0.086
Grade 10	0.172	0.341	0.373	0.113	0.160	0.348	0.374	0.118
Grade 11	0.163	0.326	0.379	0.132	0.150	0.327	0.385	0.137
Grade 12	0.132	0.306	0.401	0.160	0.134	0.310	0.395	0.161
χ^2^^*^	221,163.2				222,365.3			
*P*	<0.001				<0.001			

* *The data were tested by chi-square test for trend*.

**Figure 3:**
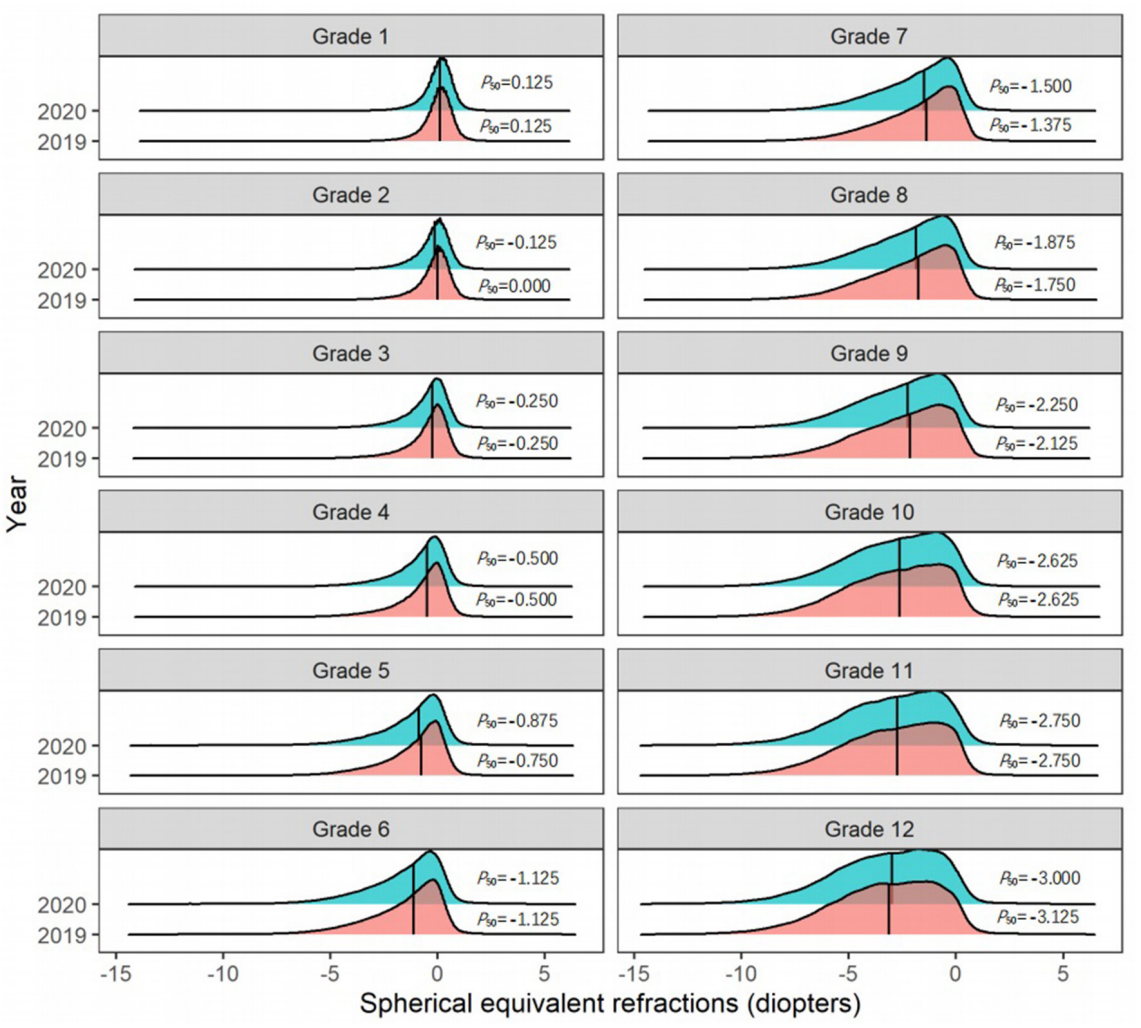
The distribution curves of spherical equivalent refraction among elementary and middle school students in 2019 and 2020.

**Figure 4:**
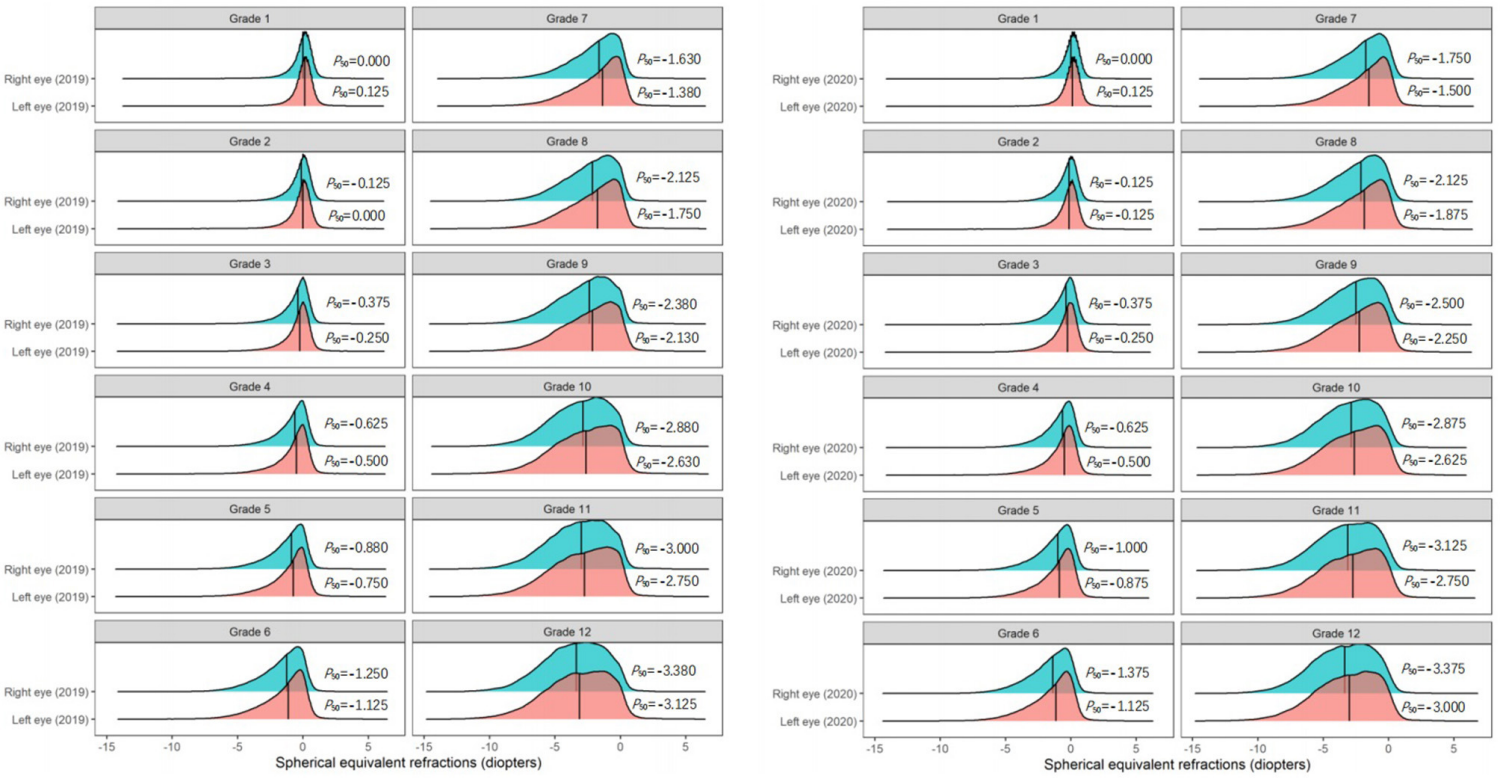
The distribution curves of spherical equivalent refraction of each eye among elementary and middle school students in 2019 and 2020.

**Figure 5:**
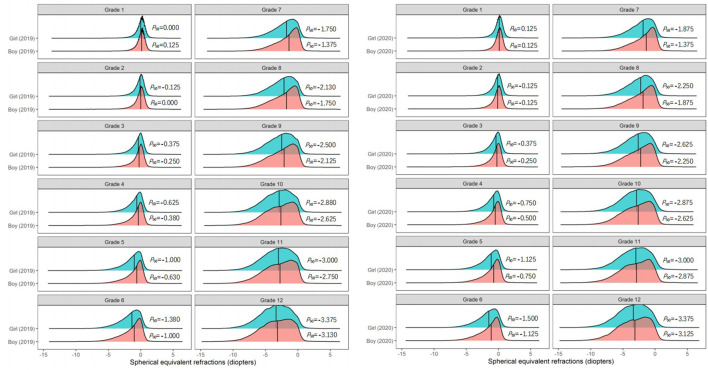
The distribution curves of spherical equivalent refraction among elementary and middle school students in 2019 and 2020 according to sex.

Among students in Grades 2 and 5 in primary schools, and Grades 7, 8, and 9 in middle schools, the *P*_50_ of the SER of the right eye in 2020 was 0.125 D lower than that in 2019 (Figure 3). Further analysis found that the *P*_50_ of SER of the right eye was lower than that of the left eye in both 2019 and 2020 (Figure 4), and that the *P*_50_ of SER of the right eye was lower among girls than that among boys in both 2019 and 2020 (Figure 5). Among girls, the *P*_50_ of the SER of the right eye decreased from −0.750 D to −0.875 D in 2020, while among boys the SER of the right eye remained unchanged at −0.625 D in 2019 and 2020 (Table 3).

**Table 3:** The quartiles of spherical equivalent refraction in the right eyes of primary and secondary school students.

**Year**	**Statistics**	**Boys**	**Girls**	U^*^	***P***
2019	*P*_25_	−1.880	−2.250	0.060	<0.001
	*P*_50_	−0.625	−0.750		
	*P*_75_	0.000	0.000		
2020	*P*_25_	−2.000	−2.375	0.061	<0.001
	*P*_50_	−0.625	−0.875		
	*P*_75_	0.000	−0.125		

* *The difference of distribution of spherical equivalent refraction between boys and girls were checked by non-parametric test*.

## Discussion

The results showed that the prevalence of myopia among elementary and middle school students in Shenzhen was 46.9% in 2019. During the COVID-19 pandemic, elementary and middle school students in Shenzhen were confined to their homes, and attended online classes. In 2020, the prevalence of myopia among these students was 50.5%, which was an 8% increase from 2019 and far exceeded the global average (17). The prevalence of myopia among elementary and middle school students in Shenzhen increased by 3.6% from 2019 to 2020, which was lower than the reported average increase of 11.7% in nine provinces in China.^1^ The possible reason for the smaller increase in Shenzhen than elsewhere in China, is that the Shenzhen government pay high attention to prevent and control myopia in children and adolescents. In 2018, 2019, and 2020, the prevalence of myopia among Chinese elementary and middle school students was 53.6 (18), 50.2^2^, and 52.7%^2^, respectively. To effectively prevent and control myopia, China issued a comprehensive implementation plan for children and adolescents in 2018, proposing to reduce the prevalence of myopia to <38% among primary school students, to <60% among middle school students, and to <70% among high school students, by 2030^3^. The Chinese government achieved positive results in 2019, but in 2020, the prevalence of myopia was close to that observed in 2018, indicating that the prevalence increased during the COVID-19 pandemic in 2020.

In recent years, with the change in lifestyle, the prevalence of myopia has also increased annually, especially in China. A 5-year longitudinal study of children and adolescents aged 6–15 years found that the prevalence of myopia increased by 10.6% annually in Chongqing (19), and a 3-year longitudinal study of children and adolescents aged 6–17 years, found that the prevalence of myopia increased by 6.3% annually in Handan (20). Consistent with previous studies (21, 22), this study found that the prevalence of myopia among elementary and middle school students increased between 2019 and 2020. The SER of the right eye decreased, and the prevalence of myopia (especially high myopia and moderate myopia) increased with increasing grade. Most of the myopic students in Grades 1–9 were assessed as having mild myopia, which is consistent with the findings of other studies (23). This study found that most of the myopic students in Grades 10–12 had moderate myopia, and that most of the myopic students in Grades 1–9 had mild myopia. One possible reason is that students had to spend more time reading and writing with each the grade increase, which may have led to loss of vision and myopia.

Li et al. (24) found that the prevalence of myopia was low before Grade 3 of primary school. A study conducted in Shandong, China found that the prevalence of myopia among primary school students in 2020 was 1.4–3 times that of the previous 5 years (23). A study in Chongqing, China, found that the prevalence of myopia among elementary and middle school students in 2020 (55.0%) was higher than that in 2019 (44.6%) (14). In the present study the prevalence of myopia among students in 2020 was 1.2, 1.1, 1.1, and 1.1 times that in 2019 in Grades 2, 3, 4, and 5, respectively. The prevalence of myopia among students in Grade 1 in 2020 was similar to that in 2019, which is consistent with previous studies (24). While the prevalence of myopia among students in Grade 12 was 0.2% lower in 2020 than in 2019, the difference was not statistically significant.

As observed in other studies (23, 25–27), in this study the prevalence of myopia in girls was higher than that in boys, and the reduction in SER in the right eyes of girls between 2019 and 2020 was higher than that in boys. Some epidemiological studies have also found that female sex is a risk factor for myopia (28). A study of the biological parameters of the ocular surface found that, girls have steeper corneas and shallower anterior chambers than boys (29). A study (30) conducted in Western China found that the progress of myopia in girls was faster than that in boys. A study (31) in Hong Kong showed that the prevalence of myopia was 37.4% in girls and 36.0% in boys, and that the progression of myopia was faster in girls than in boys. However, other studies have found no sex-specific differences in the prevalence of myopia (32, 33). In the current study, the *P*_50_ of the SER in the right eye was lower than that in the left eye. This suggests that the prevalence of myopia is higher in the right eye than the left eye, which is consistent with studies conducted in Feicheng, China (23) and Tianjin, China (34). Other studies have confirmed that the dominant eye tends to be more myopic than the non-dominant eye (35). Anisometropia is defined as unequal diopters of the two eyes. In severe cases, anisometropia may affect binocular vision (36), and the incidence of anisometropia increases with age (37). Therefore, early intervention of anisometropia (especially myopic anisometropia) in children and adolescents is very important to prevent and control myopia and improve binocular visual.

The prevalence of myopia in elementary and middle school students in areas in the former Shenzhen SEZ was higher than that in areas outside the former Shenzhen SEZ, which is similar to the findings of another study (38). Compared with areas outside the former Shenzhen SEZ, areas within it have a developed economy, high-quality educational resources, and a higher level of urbanization. Therefore, myopia may be related to economic development, education, and urbanization. The possible reasons for this are as follows: First, the former Shenzhen SEZ is more built up that the areas outside it. The four districts in the former Shenzhen SEZ account for 20.7% of the land area of Shenzhen and 31.0% of elementary and middle school students, and the density of primary and secondary school students is higher than that in the area outside the former SEZ. Second, studies have found that the urbanization process may be an environmental risk factor for the development of myopia (39); the districts in the former Shenzhen SEZ have a high level of economic development and a faster rate of urbanization. In the areas outside the former Shenzhen SEZ, the prevalence of myopia increased the most in Dapeng New District. There were no high schools in Dapeng New District until 2020, which explains the increase in the prevalence of myopia in Dapeng New District in 2020. The proportion of children in different grades remained unchanged between 2019 and 2020 in the other districts in areas outside the former Shenzhen SEZ.

This study had some limitations. First, the evaluation index of myopia selected in this study was collected using non-cycloplegic autorefraction, which may have led to the prevalence of myopia being overestimates. The human eye can self-regulate to ensure that distant and near objects can be imaged on the retina by adjusting the degree of curvature of the convex lens. In this study, cycloplegic lenses were not used to eliminate lens regulation, which may lead to inaccurate results. Second, studies have shown that myopia is caused by multiple factors, including genetic, lifestyle, material, social, and environmental factors (40, 41). This study only analyzed the prevalence and development of myopia according to region, sex, and grade at two points in time. We did not conduct a detailed analysis of the students' living habits, outdoor activities, or other factors. In the future, more specific studies should be designed to explore the factors that influence the development of myopia and the effects of intervention strategies.

In conclusion, the findings of this study suggest that home confinement during the COVID-19 pandemic may have increased the risk of myopia among elementary and middle school students, especially among students in Grades 2–5 of primary school. Younger students (particularly girls) may be more prone to develop myopia. Additional studies, including cohort studies, should be conducted to evaluate the secular trend in the development of myopia among primary and secondary school students.

## Data Availability Statement

Desensitized data can be shared with all authors of this study. Requests to access these datasets should be directed to JM, 1014120300@qq.com.

## Ethics Statement

The studies involving human participants were reviewed and approved by the Ethics Committee of Shenzhen Eye Hospital. Written informed consent to participate in this study was provided by the participants' legal guardian/next of kin.

## Author Contributions

JM, HZ, ML, MJ, XS, YC, WL, and SZ: conceptualization and methodology. JM, HZ, ML, MJ, XS, YC, and WL: data curation. JM, HZ, ML, and SZ: formal analysis. SZ: supervision. JM: visualization and writing (original draft). HZ, ML, MJ, XS, YC, WL, and SZ: writing (review and editing). All authors have approved the final version of the manuscript.

## Funding

This work was supported by Sanming Project of Medicine in Shenzhen (No. SZSM202011015).

## Conflict of Interest

The authors declare that the research was conducted in the absence of any commercial or financial relationships that could be construed as a potential conflict of interest.

## Publisher's Note

All claims expressed in this article are solely those of the authors and do not necessarily represent those of their affiliated organizations, or those of the publisher, the editors and the reviewers. Any product that may be evaluated in this article, or claim that may be made by its manufacturer, is not guaranteed or endorsed by the publisher.

## Footnotes

^1^ http://www.moe.gov.cn/fbh/live/2020/52320/mtbd/202008/t20200828_481719.html

^2^ http://www.nhc.gov.cn/xcs/s3574/202107/2fef24a3b77246fc9fb36dc8943af700.shtml

^3^ http://www.moe.gov.cn/srcsite/A17/moe_943/s3285/201808/t20180830_346672.html
